# Proportion of Racial Minority Patients and Patients With Low Socioeconomic Status Cared for by Physician Groups After Joining Accountable Care Organizations

**DOI:** 10.1001/jamanetworkopen.2020.4439

**Published:** 2020-05-08

**Authors:** Jessica T. Lee, Daniel Polsky, Robert Fitzsimmons, Rachel M. Werner

**Affiliations:** 1 Perelman School of Medicine, Division of Pulmonary, Allergy, and Critical Care, Department of Medicine, University of Pennsylvania, Philadelphia; 2 Perelman School of Medicine, Leonard Davis Institute of Health Economics, University of Pennsylvania, Philadelphia; 3 Palliative and Advanced Illness Research (PAIR) Center, Perelman School of Medicine, Department of Medicine, University of Pennsylvania, Philadelphia; 4 Johns Hopkins Bloomberg School of Public Health, Department of Health Policy and Management, Johns Hopkins University, Baltimore, Maryland; 5 Perelman School of Medicine, Division of General Internal Medicine, Department of Medicine, University of Pennsylvania, Philadelphia; 6 Center for Health Equity Research and Promotion, Corporal Michael J. Crescenz VA Medical Center, Philadelphia, Pennsylvania

## Abstract

**Importance:**

The incentive structure of accountable care organizations (ACOs) may lead to participating physician groups selecting fewer vulnerable patients.

**Objective:**

To test for changes in the percentage of racial minority patients and patients with low socioeconomic status cared for by physician groups after joining the ACO.

**Design, Setting, and Participants:**

This retrospective cohort consisted of a 15% random sample of Medicare fee-for-service beneficiaries attributed to physician groups from 2010 to 2016. Medicare Shared Savings Program (MSSP) participation was determined using ACO files. Analyses were conducted between January 1, 2019, and February 25, 2020.

**Exposures:**

Using linear probability models, we conducted difference-in-differences analyses based on the year a physician group joined an ACO to estimate changes in vulnerable patients within ACO-participating groups compared with nonparticipating groups.

**Main Outcomes and Measures:**

Whether the patient was black, was dually enrolled in Medicare and Medicaid, and poverty and unemployment rates of the patient’s zip code.

**Results:**

In a cohort of 76 717 physician groups caring for 7 307 130 patients, 16.1% of groups caring for 27.8% of patients participated in an MSSP ACO. Using 2010 characteristics, patients attributed to ACOs from 2012 to 2016, compared with those who were not, were less likely to be black (8.0% [n = 81 698] vs 9.3% [n = 270 924]) or dually enrolled in Medicare and Medicaid (12.8% [n = 130 957] vs 18.2% [n = 528 685]), and lived in zip codes with lower poverty rates (13.8% vs 15.5%); unemployment rates were similar (8.0% vs 8.5%). In the difference-in-differences analysis, there was no statistically significant change associated with ACO participation in the proportions of vulnerable patients attributed to ACO-participating groups compared with nonparticipating groups. After joining an ACO, ACO-participating groups had 0.0 percentage points change (95% CI, −0.1 to 0.1 percentage points; *P* = .59) for black patients, −0.1 percentage points (95% CI, −0.2 to 0.1 percentage points; *P* = .32) for patients dually enrolled in Medicare and Medicaid, 0.2 percentage points (95% CI, −3.5 to 4.0 percentage points; *P* = .91) in poverty rates, and −0.4 percentage points (95% CI, −2.0 to 1.2 percentage points; *P* = .62) in unemployment rates.

**Conclusions and Relevance:**

In this cohort study, there were no changes in the proportions of vulnerable patients cared for by ACO-participating physician groups after joining an ACO compared with changes among nonparticipating groups.

## Key Points

**Question:**

Is joining an accountable care organization associated with a change in the proportion of socially vulnerable patients cared for by physician groups?

**Findings:**

In this cohort study of 76 717 physician groups from 2010 through 2016, no statistically significant change was found in the percentage of racial minorities and patients with low socioeconomic status associated with participation in the Medicare Shared Savings Program.

**Meaning:**

Physician groups in general did not reduce their share of vulnerable patients after joining an accountable care organization, but continued monitoring is indicated.

## Teaser

This cohort study evaluates changes in the percentage of racial minority patients and patients with low socioeconomic status cared for by physician groups after joining accountable care organizations.

## Introduction

Accountable care organizations (ACOs) are the largest experimentation with payment reforms in the United States, with more than 1000 ACOs covering more than 32 million patients in 2018.^1^ Accountable care organizations are networks of clinicians responsible for managing the cost and quality of care for a defined population of patients across the continuum of health care settings. The largest ACO program in the country is the Medicare Shared Savings Program (MSSP). Since its implementation in 2012, the MSSP has grown to 518 ACOs caring for 10.9 million Medicare beneficiaries.^2^

Early evidence has shown ACOs have been successful in improving the quality of care and reducing costs in some cases,^3,4,5,6,7,8,9,10,11^ with nearly $800 million in shared savings reported within the MSSP in 2017.^2^ Despite these successes, there are concerns that ACOs may reinforce or potentially exacerbate disparities in health care quality, particularly by providing incentives that lead physician groups to avoid the highest-cost and most vulnerable patients.^12,13^ Prior research has demonstrated that ACOs typically form in geographic areas with fewer black residents and lower rates of poverty, fewer uninsured patients, and fewer patients without high school education.^14,15^ Additionally, ACOs that care for a higher proportion of minority patients have lower performance quality metrics.^16^ However, the evidence that ACOs avoid vulnerable patients is inconsistent. Work has demonstrated that compared with nonparticipating groups, physician groups participating in the MSSP took care of a similar proportion of patients who are racial minorities, dually enrolled in Medicare and Medicaid, or living in a high-poverty zip code^17^ and that ACOs with a high proportion of minority patients are committed to the mission of MSSP.^18^

Accountable care organizations may worsen disparities even if physician groups care for similar proportions of socially vulnerable patients when they join the ACO if, once joining, physician groups reduce the share of vulnerable patients in their panels after joining an ACO, a practice known as *cream-skimming*. Evidence has demonstrated that the highest-risk patients in terms of medical complexity and expected spending are more likely to leave ACOs, and that ACOs with more medically complex patients were more likely to drop their ACO contracting.^19,20^ Similarly, higher-cost clinicians and beneficiaries were also more likely to leave ACOs.^21^ While this supports the presence of cream-skimming based on clinical risk, to our knowledge, no prior work has examined whether ACOs are associated with cream-skimming of socially vulnerable patients. The objective of this study was to examine whether there were changes in the percentage of racial and ethnic minority patients and patients with low socioeconomic status cared for by physician groups after joining the ACO, hypothesizing that there would be a decrease in the share of vulnerable patients.

## Methods

We used a difference-in-differences framework to evaluate whether the proportions of vulnerable patients changed when physician groups joined an ACO. Changes in the proportion of vulnerable patients among physician groups that did not join an ACO were used as the comparison group in this framework. The institutional review board of the University of Pennsylvania approved this study and waived informed consent because the retrospective nature of the study made seeking informed consent infeasible and there was minimal risk to study participants. We followed the Strengthening the Reporting of Observational Studies in Epidemiology (STROBE) reporting guideline.^22^

### Data

We used 2010 to 2016 Medicare claims data for an approximately 15% random sample of Medicare fee-for-service beneficiaries to identify patients served by each physician. These claims data were supplemented with the Medicare Beneficiary Summary File for information on beneficiary enrollment, the MSSP ACO files for information on clinicians participating in the program during the study period, and the American Community Survey for zip-code–level measures of poverty and unemployment. Data analyses were conducted between January 1, 2019, and February 21, 2020.

### Study Cohort

In each year of the study period, we included all Medicare fee-for-service beneficiaries enrolled in Part A and Part B, with at least 1 primary care service from a physician in that year. Then, for each year, we attributed the beneficiaries to a physician group using the MSSP methods assigning each beneficiary annually to the physician group in which the beneficiary received the most primary care services (measured by Medicare-allowable charges).^23^ We used taxpayer identification numbers (TINs) to identify physician groups. Attribution was performed to all physician groups, regardless of whether the group participated in the MSSP. We excluded group-year observations with 10 or fewer attributed beneficiaries in a year, a step that excluded 2.2% of beneficiaries in 44.6% of physician groups.

### Variables

Our primary outcomes were 4 measures of vulnerability at the patient-year level: whether the patient was black, was dually enrolled in Medicare and Medicaid, the poverty rate of the patient’s zip code, and unemployment rate of the patient’s zip code. Our key independent variable was a time-varying indicator of whether the physician group the patient was attributed to participated in an ACO in that year. We included the following covariates in each regression: age, sex, and Charlson Comorbidity Index.^24^ We used Medicare physician claims files to identify practice size (defining small practices as having fewer than 10 physicians and large practices as having 10 or more physicians) and specialization of the practice (defining primary care practices as having 50% or more primary care physicians and specialty practices as having fewer than 50% primary care physicians). Beneficiary characteristics were identified in the first year the beneficiary was present in the data set. Physician group characteristics were calculated using means from all years the group was present in the data set.

### Statistical Analysis

Using patient-year–level data and linear probability models, we conducted difference-in-differences analyses based on the year a physician group joined an ACO to estimate changes in vulnerable patients in ACO-participating physician groups compared with changes in vulnerable patients in nonparticipating physician groups during the same period. Specifically, we estimated each outcome as a function of a time-varying indicator of whether the physician group the patient was attributed to participated in an ACO in that year, the previously mentioned covariates, and fixed effects for year and physician group. The fixed-effects method controls for unobserved differences between physician groups, allowing each physician group to serve as a control for itself. The combination of the ACO indicator variable and the fixed effects provides difference-in-differences estimates for within-physician-group changes in the outcome with ACO participation for participating groups compared with nonparticipating groups.^25,26^ All regressions accounted for clustering of patients within group practice by calculating robust standard errors. A 2-sided *P *value less than .05 was considered statistically significant. Statistical analyses were performed using Stata, version 15.0 (StataCorp).^27^

To test the sensitivity of our results to practice characteristics, we conducted stratified analyses, stratifying the full cohort by the following group characteristic: practice size, specialization of the practice, and year of ACO participation (those that joined the ACO in 2012, in 2013 or 2014, or in 2015 or 2016). We also tested the sensitivity of our results to using 2 alternative cohort definitions. First, to account for practice consolidation^28^ and physician turnover^29^ that is associated with ACO participation, we created a stable cohort of physicians and physician groups that were present every year from 2010 to 2016. We assigned each physician to the physician group in which the physician provided the plurality of services, measured by Medicare-allowable charges. Second, to account for physician groups that dropped out of an ACO, we performed an intention-to-treat analysis, coding physician groups that joined an ACO as ACO participating for the duration of the study period regardless of whether they later dropped out of the ACO.

## Results

Our cohort consisted of 76 717 physician groups caring for 7 307 130 patients, with a total of 29 111 103 patient-years. We compared patients who were attributed to ACOs between 2012 and 2016 with those who were not using their characteristics in 2010. Those who were attributed to an ACO were less likely to be black (8.0% [n = 81 698] vs 9.3% [n = 270 924]) or dually enrolled in Medicare and Medicaid (12.8% [n = 130 957] vs 18.2% [n = 528 685]) and lived in zip codes with lower poverty rates (mean proportion [SD], 13.8% [8.7%] vs 15.5% [9.1%]) (Table 1). Patients attributed to ACOs lived in zip codes with similar unemployment rates (mean proportion [SD], 8.0% [3.9%] vs 8.5% [4.3%]) compared with those not in an ACO.

**Table 1.** Characteristics of Beneficiaries in 2010 by Whether They Were Attributed to an ACO Later in the Study Period

Characteristic	Mean (SD)	
	Attributed to ACO (n = 1 024 833)	Not attributed to ACO (n = 2 912 043)
Age, y	71.5 (11.4)	72.8 (12.5)
Female, No. (%)	602 350 (58.8)	1 662 882 (57.1)
Black, No. (%)	81 698 (8.0)	270 924 (9.3)
Dually enrolled in Medicare and Medicaid, No. (%)	130 957 (12.8)	528 685 (18.2)
Poverty rate in beneficiary’s zip code	13.8 (8.7)	15.5 (9.1)
Unemployment rate in beneficiary’s zip code	8.0 (3.9)	8.5 (4.3)
Charlson Comorbidity Index score, enhanced version	1.7 (2.0)	2.1 (2.4)

Abbreviation: ACO, accountable care organization.

Most physician groups that joined an ACO were small practices (82.3% [n = 10 187]) and primary care practices (71.5% [n = 8855]) (Table 2). The number of physician groups that joined ACOs varied by year, with 2521 new ACOs in 2012 (4.9% of physician groups), 1855 in 2013 (3.7% of physician groups), 3376 in 2014 (7.0% of physician groups), 2313 in 2015 (5.0% of physician groups), and 4881 in 2016 (10.9% of physician groups). By 2016, 16.1% of physician groups had participated in an MSSP ACO and 27.8% of patients were attributed to these practices.

**Table 2.** Characteristics of Physician Groups by ACO Participation Status

Characteristic	Physician group, No. (%)^a^	
	In an ACO (n = 12 380)	Not in an ACO (n = 64 337)
Practices^b^		
Primary care	8855 (71.5)	40 901 (63.6)
Specialty	3525 (28.5)	23 436 (36.4)
Physicians in group (average size)^c^		
Small practices	10 187 (82.3)	56 394 (87.7)
Large practices	2193 (17.7)	7943 (12.4)

Abbreviation: ACO, accountable care organization.

^a^ Physician group characteristics were identified using means from all years the group was present in the data set.

^b^ Primary care practices were defined as having 50% or more primary care physicians, and specialty practices were defined as having fewer than 50% primary care physicians.

^c^ Small practices were defined as fewer than 10 physicians, and large practices were defined as 10 or more physicians.

In the difference-in-differences analysis, there were no statistically significant changes associated with ACO participation in the proportions of vulnerable patients attributed to ACO-participating physician groups compared with nonparticipating groups (Table 3). Specifically, comparing ACO-participating physician groups with nonparticipating groups, the magnitude of change after joining an ACO was 0.0 percentage points (95% CI, −0.1 to 0.1 percentage points; *P* = .59) for black patients, −0.1 percentage points (95% CI, −0.2 to 0.1 percentage points; *P* = .32) for patients dually enrolled in Medicare and Medicaid, 0.2 percentage points (95% CI, −3.5 to 4.0 percentage points; *P* = .91) in poverty rates of patient zip codes, and −0.4 percentage points (95% CI, −2.0 to 1.2 percentage points; *P* = .62) in unemployment rates of patient zip codes.

**Table 3.** Difference-in-Differences in Proportion of Vulnerable Patients Between ACO-Participating Physician Groups and Non–ACO-Participating Physician Groups

Variable^a^	Difference-in-differences estimate (95% CI)	*P* value
Black	0.0 (−0.1 to 0.1)	.59
Dually enrolled in Medicare and Medicaid	−0.1 (−0.2 to 0.1)	.32
Poverty rate of zip code	0.2 (−3.5 to 4.0)	.91
Unemployment rate of zip code	−0.4 (−2.0 to 1.2)	.62

Abbreviation: ACO, accountable care organization.

^a^ Beneficiary characteristics were identified in the first year the beneficiary was present in the data set.

Sensitivity analyses largely confirmed these findings (Table 4). While ACO participation was associated with small declines in the percentage of attributed patients who were dually enrolled in Medicare and Medicaid and in the average zip-code−level poverty rate in small physician groups and in primary care practices, in most specifications there were no statistically significant changes in vulnerable patients attributed to physician groups participating in an ACO.

**Table 4.** Sensitivity Analyses of Difference-in-Differences in Proportion of Vulnerable Patients Between ACO-Participating Physician Groups and Non–ACO-Participating Physician Groups^a^

Variable	Difference-in-differences estimate (95% CI)							
	Black	*P* value	Dually enrolled in Medicare and Medicaid	*P* value	Poverty rate of zip code	*P* value	Unemployment rate of zip code	*P* value
Physician groups^b^								
Small	0.0 (−0.1 to 0.1)	.74	−0.3 (−0.4 to −0.2)	<.001	−2.8 (−5.0 to −0.7)	.01	−0.8 (−1.8 to 0.1)	.09
Large	0.1 (−0.1 to 0.2)	.30	0.0 (−0.2 to 0.2)	.93	2.8 (−2.5 to 8.1)	.30	0.2 (−2.0 to 2.5)	.85
Practices^c^								
Primary care	0.0 (−0.1 to 0.1)	.72	−0.2 (−0.3 to −0.1)	.005	−3.6 (−6.9 to −0.3)	.03	−1.3 (−2.7 to 0.1)	.08
Specialty	0.1 (−0.1 to 0.2)	.57	0.0 (−0.3 to 0.2)	.86	2.4 (−4.1 to 8.9)	.47	0.5 (−2.2 to 3.2)	.72
Joined								
2012	0.2 (−0.1 to 0.4)	.17	0.1 (−0.1 to 0.4)	.27	4.9 (−2.6 to 12.4)	.20	1.6 (−1.5 to 4.8)	.30
2013 or 2014	−0.1 (−0.3 to 0.1)	.51	−0.2 (−0.4 to 0.1)	.18	−2.7 (−9.1 to 3.6)	.40	−1.8 (−4.5 to 0.9)	.20
2015 or 2016	−0.2 (−0.4 to 0.0)	.03	−0.1 (−0.4 to 0.2)	.64	−6.1 (−16.8 to 4.7)	.27	−2.4 (−6.0 to 1.2)	.19
Cohort								
Intention to treat^d^	0.0 (−0.1 to 0.2)	.48	0.0 (−0.2 to 0.1)	.56	−0.4 (−4.4 to 3.6)	.84	−0.7 (−2.4 to 1.0)	.43
Stable^e^	0.0 (−0.1 to 0.2)	.49	−0.1 (−0.2 to 0.1)	.50	0.5 (−3.4 to 4.4)	.80	−0.4 (−2.1 to 1.2)	.63

Abbreviation: ACO, accountable care organization.

^a^ Physician group characteristics were identified using means from all years the group was present in the data set.

^b^ Small practices were defined as fewer than 10 physicians, and large practices were defined as 10 or more physicians.

^c^ Primary care practices were defined as having 50% or more primary care physicians, and specialty practices were defined as having fewer than 50% primary care physicians.

^d^ In the intention-to-treat cohort, physician groups were coded as ACO-participating for the duration of the study period regardless of whether they later dropped out of the ACO.

^e^ The stable cohort consists of physicians and physician groups that were present every year from 2010 to 2016. We assigned each physician to the physician group in which the physician provided the most services, measured by Medicare-allowable charges.

## Discussion

We found that, overall, physician groups did not engage in cream-skimming by changing the share of socially vulnerable patients in their panels after joining an ACO. Amid reports of financial and quality successes, there has also been concern that ACOs may worsen existing disparities, at both a system and clinician level, with some evidence of selection in favor of patients with lower medical risk scores. At a clinician level, the financial incentive structure of ACOs has the potential to lead to physicians selecting fewer vulnerable patients after joining an ACO, with the expectation of higher shared savings and better performance on quality metrics.

Our findings are reassuring that this is not the case in general, although there were small changes in several subgroups examined in sensitivity analyses and differences in the panels of physician groups in 2010. Specifically, smaller physician groups and primary care practices had decreases in the share of patients dually enrolled in Medicare and Medicaid and in patients living in areas with higher poverty rates. These subgroups constituted most of the physician groups that joined ACOs. There was also a small reduction in the proportion of black patients in physician groups that joined ACOs in 2015 or 2016. These findings suggest the need for continued monitoring. While there were no changes in the proportion of vulnerable patients cared for in ACO-participating physician groups in our main analysis, there are other ways that ACOs can perpetuate or worsen disparities. Our unadjusted descriptive data showed that, based on patient characteristics in 2010, patients who were later attributed to ACOs during the study period were less likely to be black or dually enrolled in Medicare and Medicaid, and lived in zip codes with lower poverty rates. This is consistent with prior research showing that ACOs more often form in areas with fewer black residents and lower rates of poverty.^14,15^

Socially vulnerable patients may stand to benefit the most from ACOs, with prior research demonstrating worse quality of care and outcomes for patients of racial minorities or living in areas with high poverty rates.^30,31,32,33,34^ The MSSP model sets a financial benchmark for shared savings based on that population’s prior expenditures, and that may incentivize physician groups to target and keep patients with much to gain. However, it remains to be seen whether inclusion in ACOs translates to improved health outcomes in populations that have historically received worse care. Additionally, a growing minority of MSSP contracts have downside risk,^35^ meaning that ACOs that fail to meet the financial benchmark share losses. As that incentive structure becomes more common, there may be changes in physician group behavior related to vulnerable patients.

### Limitations

This study has several limitations. First, while the MSSP defines provider groups by TIN, TINs do not represent a consistent level of physician organization as some practices use a single TIN and others multiple TINs. Second, while the MSSP is the largest ACO program in the country, experiences in this population may not generalize to other ACOs. Third, we examined only 4 characteristics of social vulnerability, with limitations owing to characteristics available in the data, and both poverty and unemployment were measured at the zip code rather than patient level. Fourth, we examined a limited number of subgroups, and the overall findings may mask disparities in other subgroups, such as specific ethnicities, medical conditions, or geographic regions. Finally, because the beneficiaries who exit ACOs have higher costs and are medically higher risk,^19,20,21^ studies of the main effects of ACOs can be sensitive to whether these beneficiaries are attributed to the ACO. For example, assignment of beneficiaries to the ACO group if they were in an ACO in a particular year results in findings of modest savings, but with the potential for the high-cost beneficiaries to be in the control arm, having already exited an ACO.^36^ In contrast, an intention-to-treat assignment of beneficiaries to the ACO group if they were ever in an ACO yields results of no savings, possibly because the high-cost beneficiaries are more likely to switch clinicians and be in the ever-ACO group. We test whether the results from our study of ACO patient-shifting are sensitive to how attribution of exiting cases are handled and find no substantial sensitivity, suggesting our results are not driven by attribution method.

## Conclusions

Our findings are reassuring that physician groups did not reduce the share of vulnerable patients in their panels after joining an ACO. However, small changes in subgroups of small practices and primary care practices as well as greater prevalence of 2-sided risk models require continued monitoring for changes in behavior that would reduce access for vulnerable patient populations. Furthermore, it remains to be seen whether ACOs fulfill their promise in improving quality of care for these patients.
